# Frail phenotype is associated with distinct quantitative electroencephalographic findings among end-stage renal disease patients: an observational study

**DOI:** 10.1186/s12877-017-0673-3

**Published:** 2017-12-02

**Authors:** Chia-Ter Chao, Hsin-Jung Lai, Hung-Bin Tsai, Shao-Yo Yang, Jenq-Wen Huang, on behalf of COhort of GEriatric Nephrology in NTUH (COGENT) study group

**Affiliations:** 1 Department of Medicine, National Taiwan University Hospital Bei-Hu branch, Taipei, Taiwan; 2 Department of Medicine, National Taiwan University Hospital Jin-Shan branch, New Taipei City, Taiwan; 3 Graduate Institute of Toxicology, School of Medicine, National Taiwan University, Taipei, Taiwan; 4 Department of Internal Medicine, National Taiwan University Hospital, NO.7, Chung-Shan South Road, Zhong-Zheng district, Taipei, 100 Taiwan; 5 Community and Geriatric Medicine Research Center, National Taiwan University Hospital BeiHu branch, Taipei, Taiwan; 6 Department of Neurology, National Taiwan University Hospital and College of Medicine, Taipei, Taiwan

**Keywords:** Chronic kidney disease, Electroencephalogram, End-stage renal disease, Frail phenotype, Frailty, Neurophysiological monitoring, Simple FRAIL scale

## Abstract

**Background:**

Frailty is prevalent among patients with end-stage renal disease (ESRD) and is associated with an increased risk of cognitive impairment. However, apart from its influence on cognition, it is currently unknown whether frailty affects subtler cerebral function in patients with ESRD.

**Methods:**

Patients with ESRD were prospectively enrolled, with clinical features and laboratory data recorded. The severity of frailty among these patients with ESRD was ascertained using the previously validated simple FRAIL scale, and was categorized as none-to-mild and moderate-to-severe frailty. All participants underwent quantitative electroencephalography (EEG), with band powers documented following the generation of the delta to alpha ratio (DAR) and delta/theta to alpha/beta ratio (DTABR). EEG results were then compared between groups of different levels of frailty.

**Results:**

In this cohort, (mean age: 68.9 ± 10.4 years, 37% male, 3.4 ± 3 years of dialysis), 20, 60, 40, 17, and 6% patients exhibited positivity in the fatigue, resistance, ambulation, illness, and loss-of-body-weight domains, respectively, with 45.7% being none to mildly frail and 54.3% being moderately to severely frail. Those with mild frailty had a significantly higher delta power compared to those with more severe frailty, involving all topographic sites. Patients with ESRD and severe frailty had significantly lower global, left frontal, left temporo-occipital, and right temporo-occipital DAR and DTABR, except in the right frontal area, and tended to have central accentuation of alpha, beta, and theta power, and more homogeneous DTABR and DAR distribution compared to the findings in those with mild frailty.

**Conclusions:**

Frailty in patients with ESRD can have subtler neurophysiological influences, presenting as altered EEG findings, which warrant our attention.

## Background

Frailty is a geriatric syndrome, which is characterized by a diminished physiological reserve to tackle external stressors and the accumulation of subtle deficits across multiple organ systems. Patients with chronic kidney disease (CKD), particularly end-stage renal disease (ESRD), exhibit a higher prevalence of frailty (i.e., 14–20% and 30–60%, respectively), which is at least 3–8-fold higher than that in the healthy, community-dwelling elderly population [1–4]. CKD has now been recognized as a state of accelerated aging, owing to mechanisms involving allostatic load increase, the activation of stress-resistance response, and an impaired anti-aging pathway [5]. Thus, it is expected that an aging-related phenotype, including frailty, will be more prevalent in patients with renal insufficiency. Emerging studies further suggest that patients with ESRD, who also have diabetes, hypoalbuminemia, increased inflammation, or are hospitalized are more likely to manifest worsening frailty over time, compared with those without these symptoms [6]. Frailty may also be an important, yet under-recognized opportunity, to avert the adverse consequences, which arise due to unsuccessful aging in patients with CKD [7].

The presence of frailty in patients with CKD correlates with a diverse spectrum of negative health outcomes. Frailty confers a significantly higher risk of mortality among patients with cardiovascular illnesses. Patients undergoing chronic dialysis with frailty also have aberrant cardiac conduction and a higher risk of shunt failure [8–10]. In addition, the “frail dialysis phenotype” or “frail renal phenotype” has been coined by researchers to describe those with ESRD and frailty at the same time [11, 12]; affected patients may have metabolic and musculoskeletal sequel compared to their non-frail counterparts, manifesting as an increased risk of osteopenia, vertebral compression fracture, and altered body fat distribution in the former group [13, 14]. Recently, the neurological effects of frailty were gradually recognized in these patients as well. McAdams-DeMarco et al. [15] reported that among patients with incident ESRD, frailty was independently associated with poorer cognitive function at baseline and during follow-up 1 year later. Apart from cognitive impairment, however, other aspects of cerebral function affected by frailty are rarely examined in patients with ESRD.

The electroencephalogram (EEG), which is generated from electrodes placed upon specific areas over the scalp and the aggregation of postsynaptic potentials with amplification, acts as a footprint of cortical function [16]. Owing to its simplicity and portable nature, the EEG has been widely utilized for detecting and characterizing seizure in various settings, guiding therapeutic decisions during neurosurgery or anesthesia, and for prognostication in neuropsychiatric illnesses [16–18]. Quantitative EEG analysis, an objective summary of the visual EEG tracings, further allows clinicians to evaluate alterations in cortical activities earlier, and its findings are found to correlate strongly with clinical progression in many neurological diseases [19, 20]. In the current pilot study, we hypothesized that frailty might be associated with neurophysiological changes in patients with ESRD under chronic dialysis, an issue that has not been addressed in the literature previously. Through the assistance of quantitative EEG, we aimed to examine the differences in cortical activities from patients with ESRD, based on their levels of frailty, and explore the clinical implications.

## Methods

### Study participants and clinical characteristics documentation

Patients with ESRD under chronic hemodialysis were prospectively enrolled from National Taiwan University Hospital Jinshan branch, according to previously published procedures [9, 14]. In brief, we included patients with ESRD under chronic dialysis with catastrophic illness certificates, which served as a validated proof of their chronic dialysis status. The exclusion criteria included refusal to participate and pregnancy; we excluded pregnant participants at the time of screening due to their vulnerability to injuries in clinical studies. On enrolment, patients were interviewed and their clinical information, including age, gender, relevant comorbidities, and body mass index (BMI), was recorded. We recorded comorbidities with particular emphasis on those potentially having neurologic impacts, including hypertension, diabetes, cirrhosis, heart failure, and malignancy, based on compatible medical histories and appropriate image or laboratory examinations. Blood specimens were collected during the monthly blood tests, subjecting to a complete hemogram and biochemical analysis, including serum urea nitrogen and creatinine, nutritional parameters (albumin, total cholesterol, triglyceride, low and high density lipoprotein), electrolyte panels (sodium, potassium, chloride, calcium, and phosphate), and dialysis adequacy (Kt/V, urea-reduction ratio). Concurrent medications with influences on consciousness, such as benzodiazepine, anti-depressants, and anti-psychotics, were also recorded.

The state of frailty was ascertained using the simple frail scale (SFS), which has been repeatedly validated for optimal application in different populations, including patients with ESRD [21, 22]. The SFS uses five self-reported questions to examine five important components of frailty, namely Fatigue, Resistance, Ambulation, Illness, and Loss of body weight (leading to the acronym “FRAIL”). Each question is assigned one point, and the score for each patient is calculated by the total of all five questions; patients with high scores (up to 5) had severe frailty. Patients with SFS scores of 0 and 1 were categorized as none to mildly frail, while those with a score of 2 or higher were categorized as moderately to severely frail, to balance the case numbers in each group. The investigators and nurse researchers administered the SFS twice during the inter-dialytic period, and the average scores were obtained to confirm the consistency of assessment results.

### Electroencephalographic data acquisition and analysis

During the intra-dialytic period, patients with ESRD were maintained awake in a seated or prone position, with eyes closed. A total of 21 silver/silver chloride cup electrodes were placed on the scalp, as per International 10-20 system. Signals were amplified, digitalized, and stored using a 32-channel digital EEG system (Neurofax EEG 2100, Nihon Kohden Corp, Tokyo, Japan). EEG data were reprocessed using a band-pass filter with cut-off frequencies of 1 Hz and 32 Hz and resampled to 64 Hz to reduce computational complexity. EEG signals with simultaneous amplitudes >150 μV were deemed contaminated and trimmed. The initial 2 min of the processed data were extracted for measurement of band power. The EEG signals from every sampled lead were subjected to a 3-level wavelet transformation and were decomposed into the following frequency bands: delta (<4 Hz), theta (4–7 Hz), alpha (8–15 Hz), and beta (16–30 Hz), grouped according to the cerebral topography that is widely used (i.e., frontal left [FL] group, Fp1, F3, and F7; frontal right [FR] group, Fp2, F4, and F8; Central [Z] group, Fz, Cz, and Pz; temporo-occipital left [TL] group, C3, P3, T3, T5, and O1; temporo-occipital right [TR] group, C4, P4, T4, T6, and O2) [17]. The band power was also used to calculate the delta to alpha ratio (DAR) and delta/theta to alpha/beta ratio (DTABR), which was then compared between groups.

### Statistical analysis

Continuous variables were expressed as mean ± standard deviation and compared between groups using a Student’s *t*-test or one-way analysis of variance (ANOVA). Categorical variables were expressed as percentages and compared between groups using a chi-square test. We first compared the clinical features (demographic and physical parameters, comorbidities, laboratory data, and chronic medications) between those with none to mild frailty (SFS score: 0–1) and those with moderate to severe frailty (SFS score: 2–5). Thereafter, we compared EEG band power parameters, including the DTABR and DAR, between the two groups (none to mild vs. moderate to severe), and between patients with ESRD of different frailty. The DTABR and DAR are established quantitative EEG parameters, which exhibit prognostic importance for patients with different types of neuropsychological illnesses [23, 24]. Spectral analysis with brain EEG mapping for the different frequency bands was also done to evaluate anatomical clustering. A topographic illustration of band power distribution was provided for those with characteristic quantitative EEG presentations.

### Ethical approval

The current study was approved by the ethics committee of National Taiwan University Hospital (NO.201403006RINB). Verbal informed consent was obtained from all participants.

## Results

In total, 36 patients were screened during the initial phase; 1 patient refused to participate, leaving 35 patients enrolled for subsequent analysis. The mean age of the study cohort was 68.9 ± 10.4 years (Table 1). Approximately 37% of the study cohort were men and the average dialysis experience and BMI were 3.4 ± 3 years and 23.5 ± 4 kg/m^2^, respectively. Overall, the most common comorbidity was hypertension (86%), followed by diabetes mellitus (DM; 51%), heart failure (23%), and malignancy (9%).

**Table 1:** Clinical features of enrolled ESRD patients with different frail severity

Variables	None to mildy frail (*n* = 16; 45.7%)	Moderately to severely frail (*n* = 19; 54.3%)	*p* value
*Demographic and physical features*			
Age (years)	65.1 ± 11.3	72.1 ± 8.7	*0.05*
Gender (male %)	8 (56)	4 (21)	*0.03*
Body mass index (kg/m^2^)	23.9 ± 4.2	23.1 ± 3.9	*0.58*
Dialysis duration (years)	2.4 ± 1.7	4.3 ± 3.6	*0.06*
*Comorbidities*			
Hypertension (%)	14 (88)	16 (84)	*0.79*
Diabetes (%)	7 (44)	11 (58)	*0.42*
Liver cirrhosis (%)	1 (6)	2 (11)	*0.67*
Heart failure (%)	3 (19)	5 (26)	*0.61*
Malignancy (%)	2 (13)	1 (5)	*0.46*
*Laboratory data*			
Hemoglobin (g/dL)	10.1 ± 1.3	9.2 ± 1.1	*0.02*
Leukocyte count (K/μL)	6.5 ± 1.5	7.9 ± 2.9	*0.09*
Platelet (K/μL)	185 ± 38	208 ± 62	*0.2*
Albumin (mg/dL)	3.8 ± 0.3	3.7 ± 0.3	*0.29*
Urea nitrogen (mg/dL)	79.1 ± 20.3	78.2 ± 18.2	*0.88*
Sodium (meq/L)	135 ± 3.9	136 ± 3.1	*0.47*
Creatinine (mg/dL)	11.6 ± 2.2	9.9 ± 1.8	*0.01*
Potassium (meq/L)	4.8 ± 0.7	4.9 ± 0.7	*0.63*
Calcium (meq/L)	9 ± 0.7	9 ± 0.8	*0.86*
Phosphate (meq/L)	5.3 ± 1.3	5 ± 1.4	*0.44*
Chloride (meq/L)	99.4 ± 4.1	100.2 ± 3.4	*0.54*
Total cholesterol (mg/dL)	159 ± 37	167 ± 54	*0.62*
Triglyceride (mg/dL)	149 ± 102	172 ± 112	*0.53*
High-density lipoprotein (mg/dL)	41 ± 9	39 ± 13	*0.64*
Low-density lipoprotein (mg/dL)	96 ± 30	102 ± 37	*0.57*
*Dialysis-related parameters*			
Kt/V	1.55 ± 0.2	1.69 ± 0.13	*0.02*
Urea reduction ratio (%)	72.5 ± 5	76.2 ± 2.5	*0.01*
*Chronic medication use*			
Benzodiazepine (%)	9 (56)	6 (32)	*0.15*
Anti-depressants (%)	5 (31)	2 (11)	*0.13*
Anti-psychotics (%)	0 (0)	2 (11)	*0.19*

Data are expressed as mean ± standard deviation for continuous variable and number (percentage) for categorical variables

*ESRD* end-stage renal disease

Using SFS as previously described [14, 21, 25], we found that 20, 60, 40, 17, and 6% patients were positive for fatigue, resistance, ambulation, illness, and loss-of-body-weight domains, respectively, leading to 45.7% (*n* = 16) and 54.3% (*n* = 19) of the participants being categorized as none to mildly frail and moderately to severely frail, respectively (Table 2). Patients who were moderately to severely frail had a borderline higher age (*p* = 0.05) and were more likely to be women (*p* = 0.03), compared to those who were none to mildly frail, without differences in BMI, durations of dialysis, or prevalence of comorbidities that were examined (Table 1). Patients who were moderately to severely frail had significantly lower levels of hemoglobin (*p* = 0.02) and serum creatinine (*p* = 0.01), but higher dialysis clearance rates (Kt/V and urea reduction ratio), compared to those who were mildly frail.

**Table 2:** Frailty assessment results in this cohort

Components	Total (*n* = 35)	None to mildy frail (*n* = 16; 45.7%)	Moderately to severely frail (*n* = 19; 54.3%)	*p* value
Fatigue (%)	7 (20)	1 (6)	6 (32)	*0.07*
Resistance (%)	21 (60)	2 (13)	19 (100)	*< 0.01*
Ambulation (%)	14 (40)	1 (6)	13 (68)	*< 0.01*
Illness (%)	6 (17)	0 (0)	6 (32)	*0.01*
Body weight loss (%)	2 (6)	0 (0)	2 (11)	*0.19*

A comparison of all the quantitative EEG findings between participants with different severity of frailty is shown in Table 3. Although there were no significant differences in terms of the alpha, beta, and theta waves between participants with and without frailty, participants with mild frailty had a significantly higher delta wave power, at all topographical sites, than those with severe frailty (*p* = 0.03 for right frontal region; *p* = 0.01 for central and right TO region; *p* < 0.01 for left TO and frontal region).

**Table 3:** Quantitative electroencephalographic results in this cohort

EEG variables	None to mildly frail (*n* = 16; 45.7%)	Moderately to severely frail (*n* = 19; 54.3%)	*p* value
*Alpha wave*			
Central	24.2 ± 19.9	20.2 ± 12.2	*0.58*
Right temporo-occipital	17.4 ± 17.1	16.3 ± 10.3	*0.82*
Left temporo-occipital	24.7 ± 16.9	25 ± 16.6	*0.97*
Right frontal	20.8 ± 16.6	19 ± 12.7	*0.73*
Left frontal	24.2 ± 19.9	20.2 ± 12.2	*0.5*
*Beta wave*			
Central	9.3 ± 5.1	10.5 ± 10.8	*0.71*
Right temporo-occipital	5.5 ± 4.2	7.7 ± 7.2	*0.32*
Left temporo-occipital	8.3 ± 5.9	10.3 ± 11.6	*0.55*
Right frontal	9.7 ± 8.1	10 ± 10.1	*0.93*
Left frontal	12.6 ± 9.6	12.7 ± 14.5	*0.98*
*Delta wave*			
Central	49,967 ± 77,553	1540 ± 2363	*0.01*
Right temporo-occipital	34,693 ± 56,300	1158 ± 2002	*0.01*
Left temporo-occipital	44,802 ± 66,187	1735 ± 2843	*< 0.01*
Right frontal	44,333 ± 69,328	6057 ± 20,077	*0.03*
Left frontal	61,060 ± 87,196	1382 ± 2572	*< 0.01*
*Theta wave*			
Central	36.4 ± 31.1	27.9 ± 25.8	*0.39*
Right temporo-occipital	16 ± 16.1	13.1 ± 12.7	*0.56*
Left temporo-occipital	19.6 ± 17.8	17.3 ± 13.2	*0.67*
Right frontal	24.3 ± 17.3	22.1 ± 21.6	*0.76*
Left frontal	26.5 ± 20.2	22.5 ± 20.8	*0.59*

Data are expressed as mean ± standard deviation for continuous variable and number (percentage) for categorical variables

**Fig. 1:**
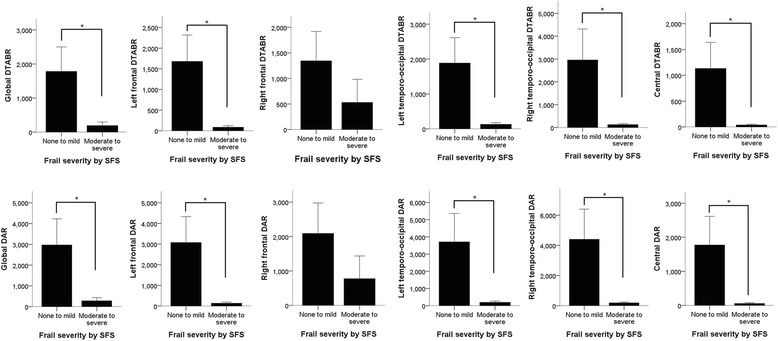
Comparison of electroencephalographic parameters (delta-alpha ratio [DAR] and delta-theta-to-alpha-beta ratio [DTABR]) between patients with ESRD with different frailties based on topographical areas. ESRD, end-stage renal disease; SFS, simple FRAIL scale

We further analyzed the global and regional DAR and the DTABR indices among patients with none to mild frailty and moderate to severe frailty. It is noteworthy that patients undergoing dialysis with moderate to severe frailty had significantly lower global DAR (the former vs. the latter, 283 ± 679 vs. 2971 ± 4859, *p* = 0.02), and DARs of the left frontal (135 ± 250 vs. 3073 ± 4702, *p* = 0.01), left TO (197 ± 318 vs. 3708 ± 6398, *p* = 0.02), central (55 ± 96 vs. 1773 ± 3262, *p* = 0.03), and right TO (187 ± 261 vs. 4400 ± 7763, *p* = 0.02), compared to those with none to mild frailty, except in the right frontal area (Fig. 1). Similarly, those with moderate to severe frailty had significantly lower global DTABR (the former vs. the latter, 191 ± 469 vs. 1781 ± 2793, *p* = 0.02), and DTABRs of the left frontal (86 ± 158 vs. 1680 ± 2388, *p* < 0.01), left TO (130 ± 210 vs. 1884 ± 2828, *p* = 0.01), central (39 ± 65 vs. 1132 ± 1957, *p* = 0.02), and right TO DTABR (126 ± 178 vs. 2960 ± 5271, *p* = 0.03) than those with none to mild frailty, except in the right frontal area (Fig. 1). A sensitivity analysis through sub-dividing patients according to SFS scores showed that global DTABR index declined progressively with increasing frailty (i.e., 0 (1448 ± 2793) vs. 1 (3111 ± 2895) vs. 2 (14 ± 15) vs. 3 (252 ± 560) vs. 4 (143 ± 162)).

**Fig. 2:**
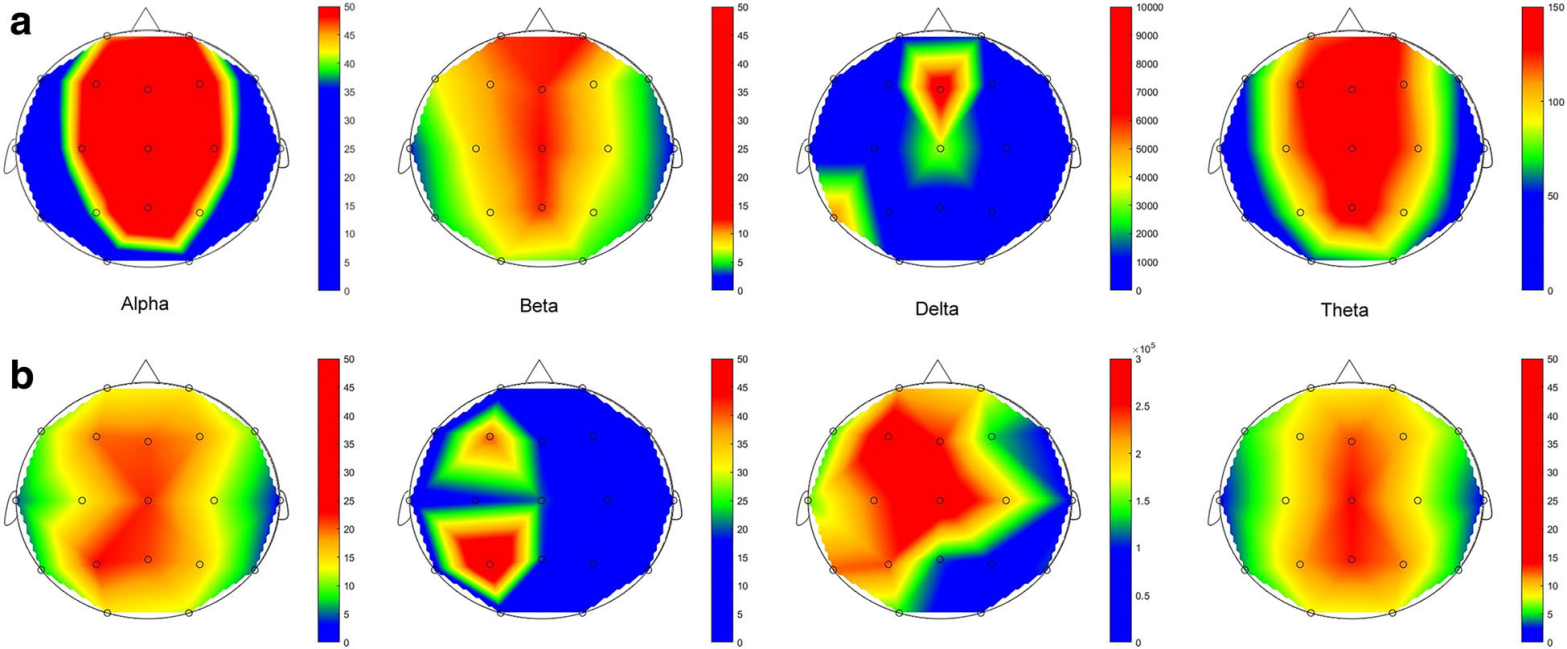
Representative topographical images from two patients with ESRD, one with moderate to severe frailty (**a**, upper row) and the other with none to mild frailty (**b**, lower row), based on band powers of different frequencies. ESRD, end-stage renal disease

**Fig. 3:**
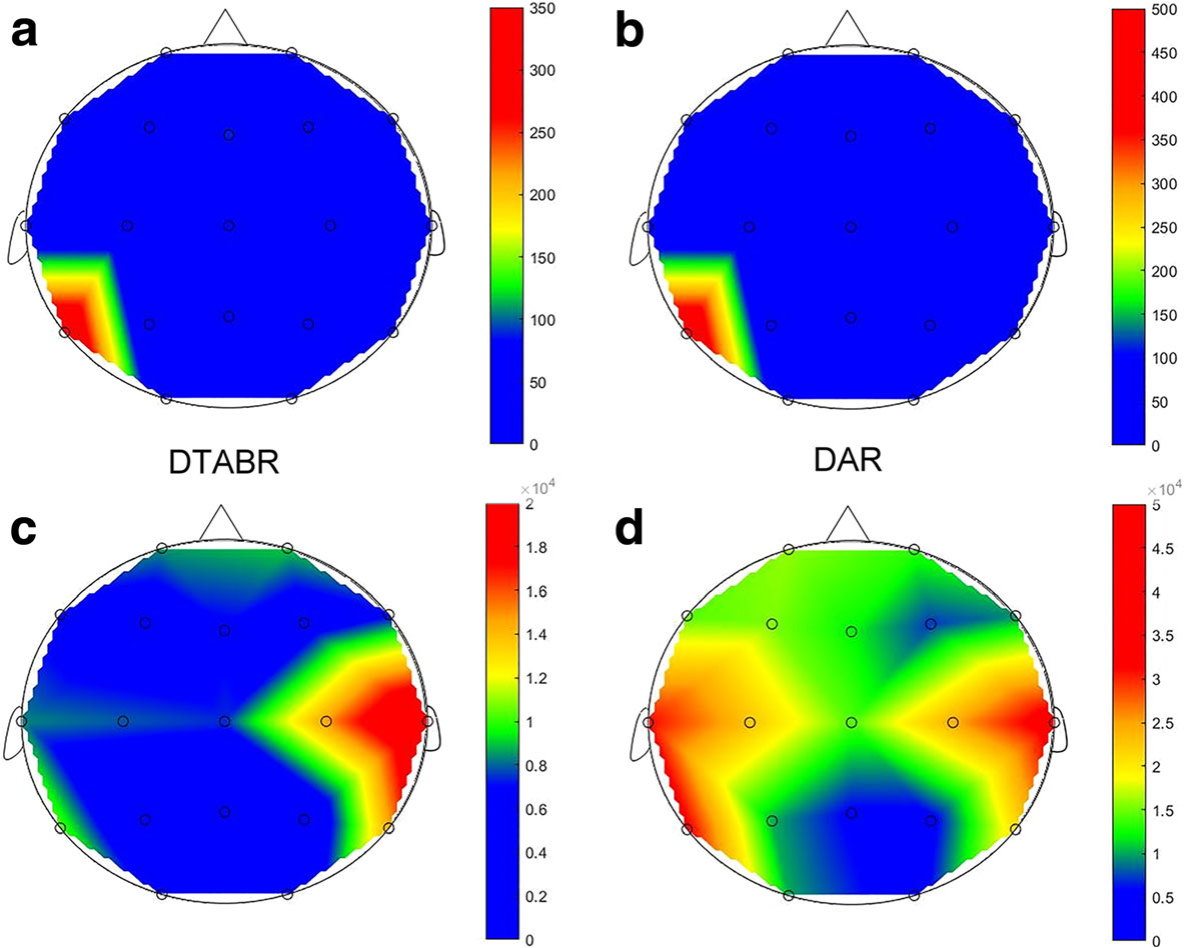
Representative topographical images from the same candidates with ESRD in Fig. 2, one with moderate to severe frailty (upper row; based on DTABR [**a**] and DAR [**b**]) and the other with none to mild frailty (lower row; based on DTABR [**c**] and DAR [**d**]). DAR, delta-alpha ratio; DTABR, delta-theta-to-alpha-beta ratio

Typical cerebral topography images, which were constructed based on EEG band powers of individual dialysis patients with mild and severe frailty, are shown in Figs. 2 and 3, respectively. Those with severe frailty tend to have central or central-and-bi-frontal accentuation of alpha, beta, and theta waves compared to those with mild frailty, while the latter group tended to have lateralization of alpha, beta, and delta waves (Fig. 2). In addition, participants with severe frailty tended to have generalized lower DTABR and DAR indices. Further, their signals were more homogenous compared with those with mild frailty (Fig. 3).

## Discussion

Among a cohort of patients with ESRD under chronic dialysis receiving quantitative EEG examinations, we found that those with none to mild frailty had significantly higher delta wave power in all tested areas. This difference resulted in significantly higher DTABR and DAR indices involving most areas except the right frontal one. In addition, a tendency of band power lateralization was observed among those with none to mild frailty.

Few studies in the literature focus on the quantitative EEG findings in patients with CKD/ESRD. Sagales et al. [26] utilized quantitative EEG to illustrate the differences in brain function between patients with uremia and healthy participants, in a pilot study. They found that all the band powers in patients with uremia were more significantly attenuated than those in healthy participants. The alpha-predominance pattern decreased while delta wave accentuation by optic stimulation was amplified in the former group. The distribution pattern of different frequencies (lower frequencies in frontal areas and higher frequencies in central and TO areas) was also lost in patients with uremia [26]. The current study also corroborated with these findings since we also discovered a striking elevation in delta wave power, diffusely (Table 3). Further, the overall DAR and DTABR indices were 10–100-fold higher among patients with ESRD, compares with those with non-renal illnesses [24, 27] and healthier ones (less than 1) [28]. These results indirectly suggested a generalized suppression of alpha power. Similar findings of significant alpha and beta suppression with delta accentuation were also described in a recent study done in stable patients with CKD and ESRD [29], lending support to the validity of our results.

The role quantitative EEG plays in outcome prediction has attracted much attention recently, particularly due to the utilization of novel parameters such as DAR and DTABR. In patients with non-acute brain acquired injury, higher DAR reportedly predicted less physical improvement after rehabilitation, while other EEG parameters do not [30]. Further, a higher DAR was associated with worsened daily activity status following a stroke or major cerebral trauma [24, 31]. Similarly, the presence of higher DTABR in patients with ischemic anterior and posterior syndrome was associated with a larger infarct size, and unfavorable short-term and long-term neurologic recovery [23, 27]. However, existing studies looking at these novel EEG parameters mostly included patients with cerebral ischemia and injury. None of the studies address patients with frailty, which is a more indolent process that is frequently accompanied by an increased incidence of cerebral dysfunction. In the current study, patients with ESRD had, on average, significantly higher DAR and DTABR, compared with healthy controls and those without renal illnesses in the literature. A plausible reason for this phenomenon might be the impaired performance observed in patients with CKD/ESRD [32, 33], similar to those observed in patients sustaining cerebral insults. Furthermore, we discovered that DAR and DTABR rose substantially among patients with ESRD with none or mild frailty, which is a finding that has not been described before. This result can be attributed to several reasons. First, patients with none or mild frailty had significantly higher levels of hemoglobin, compared with those with moderate to severe frailty (Table 1); thus, those from the former group received a lower dosage of erythropoietin than the latter (4500 U/week vs. 8500 U/week epoetin beta). Since anecdotal reports indicates that erythropoietin can restore EEG abnormalities observed in patients with ESRD [26], patients with none to mild frailty receiving a smaller dose of erythropoietin, would develop more prominent EEG abnormalities such as an striking increase in delta power, DAR, and DTABR. In addition, patients with none to mild frailty in the current study had significantly lower dialysis adequacy (Kt/V) compared to those with moderate to severe frailty (Table 1), rendering the former group susceptible to the unfavorable neurologic sequel brought by uremia. Past reports suggested that patients with CKD tend to have more prominent delta wave activities with worsening renal function due to uremic encephalopathy [34], resulting in the observed increase in delta power among those with mild frailty in the current study. Finally, depression or anxiety reportedly influences delta power in affected patients [35, 36]. Given the increased prevalence of depression or anxiety in patients with ESRD and frequent co-existence of frailty with these disorders [37, 38], it is possible that the observed EEG patterns can be attributed to the discrepancy in the prevalence of these disorders between patients with different frail severity. However, further study, with a larger cohort, is required to validate our findings.

## Limitations

The current study has multiple limitations that should be acknowledged. First, given the low case number, statistical limitations apply. However, judging from the striking discrepancies in the EEG parameters (i.e., DAR and DTABR) between patients with ESRD with different frail severities, we believe that there is a real association between frailty and these novel EEG parameters. Another limitation of the current study is that we did not assess the prevalence of psychiatric disorders in the patient cohort. Finally, we did not evaluate the cognition function in the patient cohort using instruments such as the Mini-mental Status Examination or Trail Making Test. Thus, the exact underlying mechanisms contributing to the relationship observed remains unknown. Nonetheless, since this is a pilot study, we intend to conduct a larger study in the future to confirm our findings.

## Conclusions

In conclusion, using a well-validated modest-sized ESRD cohort, we found that those with none to mild frailty had significantly higher delta power, DAR, and DTABR on quantitative EEG, compared with the delta power in those with moderate to severe frailty. This phenomenon has not been previously described. Our results suggest that frailty in patients with ESRD can have subtler neurophysiologic influences other than cognitive dysfunction, paving the way toward a more in-depth understanding of its pathophysiological role.

## Abbreviations

ANOVA: One-way analysis of variance
BMI: Body mass index
CKD: Chronic kidney disease
DAR: Delta-to-alpha ratio
DTABR: Delta/theta to alpha/beta ratio
EEG: Electroencephalogram
ESRD: End-stage renal disease
FL: Frontal left
FR: Frontal right
SFS: Simple FRAIL scale
TL: Temporo-occipital left
TR: Temporo-occipital right
